# Supplementary material for: Mask - Region-based Convolutional Neural Networks (R-CNN) with Radiomics Integration and Gray Level Co-occurrence Matrix (GLCM) for brain tumor detection and segmentation
Source: PLoS One. 2026 Feb 23;21(2):e0342185. doi: 10.1371/journal.pone.0342185 (PMC12928456; doi:10.1371/journal.pone.0342185)
Supplement: Attachment — Submitted filename: Prathima review comments.docx [file pone.0342185.s002.docx]

Additional Editor Comments (if provided):

Author presented the research entitled “Mask - Region-based Convolutional Neural Networks (R-CNN) with Radiomics Integration and Gray Level Co-occurrence Matrix (GLCM) for brain tumor detection” well. Here I would like to point out some suggestion to proceed further.

The manuscript should specify dataset sources (e.g., Figshare, Br35H, BraTS), image count, resolution, and data partition ratios (training/testing).

Response: Thank you for your comment. The manuscript has been updated to clearly specify the dataset sources (Figshare and Harvard Whole Brain Atlas), including image count, resolution, and data partition ratios for training, validation, and testing.

Clarify pre-processing techniques—normalization, augmentation, and noise reduction—to ensure reproducibility.

Response: Thanks for your comment. We have added the pre-processing techniques section, which now includes details on **normalization**, **augmentation**, and **noise reduction**. These steps are critical for ensuring reproducibility across experiments.

The paper mentions radiomics and GLCM integration, but the fusion mechanism (concatenation, attention weighting, or dimensionality reduction) is not clearly explained.

Response: Thanks for your comment. We have added the Feature Fusion Mechanism section

Add an ablation study to show the contribution of each component (Mask R-CNN, radiomics, GLCM).
Ensure consistency in terminology—use either “brain tumor classification” or “detection and segmentation” throughout the manuscript.
Response: Thanks for your comment. We have added an ablation study to demonstrate the contribution of each component—Mask R-CNN, radiomics, and GLCM—to the overall model performance. The corresponding results and discussion have been included in the revised manuscript. Additionally, we have ensured consistency in terminology throughout the manuscript by uniformly using the term “brain tumor detection and segmentation.

Add recent literature references (2022–2025) comparing hybrid deep learning and radiomics models for medical imaging.

Response: Thank you for your insightful comment. Recent 2022–2025 references added.

Include a “Limitations and Future Work” section discussing scalability, dataset diversity, and model generalizability
Response: Thank you for your valuable comment. A section on Limitations and Future Work has been added after the Conclusion. This section addresses the challenges related to scalability, dataset diversity, and the generalizability of the model, and outlines potential directions for future research.

Reviewers' comments:

Reviewer's Responses to Questions

**Comments to the Author**

1. Is the manuscript technically sound, and do the data support the conclusions?

The manuscript must describe a technically sound piece of scientific research with data that supports the conclusions. Experiments must have been conducted rigorously, with appropriate controls, replication, and sample sizes. The conclusions must be drawn appropriately based on the data presented.

Reviewer #1: Yes

2. Has the statistical analysis been performed appropriately and rigorously?

Reviewer #1: Yes

3. Have the authors made all data underlying the findings in their manuscript fully available?

The [PLOS Data policy](http://www.plosone.org/static/policies.action#sharing) requires authors to make all data underlying the findings described in their manuscript fully available without restriction, with rare exception (please refer to the Data Availability Statement in the manuscript PDF file). The data should be provided as part of the manuscript or its supporting information, or deposited to a public repository. For example, in addition to summary statistics, the data points behind means, medians and variance measures should be available. If there are restrictions on publicly sharing data—e.g. participant privacy or use of data from a third party—those must be specified.

Reviewer #1: Yes

4. Is the manuscript presented in an intelligible fashion and written in standard English?

PLOS ONE does not copyedit accepted manuscripts, so the language in submitted articles must be clear, correct, and unambiguous. Any typographical or grammatical errors should be corrected at revision, so please note any specific errors here.

Reviewer #1: Yes

5. Review Comments to the Author

Please use the space provided to explain your answers to the questions above. You may also include additional comments for the author, including concerns about dual publication, research ethics, or publication ethics. (Please upload your review as an attachment if it exceeds 20,000 characters)

Reviewer #1: The manuscript proposes a new technique called “Mask - Region-based Convolutional Neural Networks (R-CNN) with Radiomics Integration and Gray Level Co-occurrence Matrix (GLCM) for brain tumor detection”. This is a research project with scientific and practical significance. The manuscript is presented with a reasonable layout and rich simulation results, demonstrating the effectiveness of the proposed method. To improve the quality of the manuscript, the following ideas should be considered for editing.

Review Comments:
1. Need to change the “the offering of the our work are follows” in the introduction section.

Response: Thank you for your comment. We have revised the phrase “the offering of our work are follows” to “the contributions of our work are as follows” in the Introduction section.

2. Abbreviations only need explanation the first time they are used. There are a number of abbreviations that are explained multiple times in the manuscript. For example, CNN is explained twice in the literature.

Response: Thank you for your comment. We have carefully reviewed the manuscript and ensured that each abbreviation is defined only at its first occurrence (e.g., CNN, MRI, MLP). Repeated explanations have been removed for consistency and clarity.

3. Limited literature review has been carried out, so limitations of existing works are not reflected.

Response: Thank you for your comment. We have added the Critical Review and Observed Limitations of Prior Studies and corrected limitations of exiting model.

4. How does the R2CNN architecture improve tumor region localization compared to standard CNN models?

Response: Thank you for your comment. We have added the content in section Improvement over Standard CNN Architectures and Localization Advantage of Mask R-CNN (R2CNN).

5. Many of the equations are part of the related sentences. Attention is needed for correct sentence formation.

Response: Thank you for your comment. We have carefully reviewed the manuscript and revised the related sentences to ensure that all equations are correctly integrated within their corresponding textual context for improved readability and grammatical accuracy.

6. What role do radiomics features play in enhancing the detection accuracy of brain tumors?

Response: Thank you for your comment. Radiomics features quantitatively capture tumor texture, shape, and intensity variations, enhancing the model’s ability to identify subtle heterogeneity and improving the accuracy of brain tumor detection.

7. The manuscript does not discuss potential limitations such as scalability, real-world applicability, or robustness to unseen data.

Response: Thank you for your comment. We appreciate this valuable observation. The revised manuscript now includes a dedicated section titled “Limitations and Future Work”, which explicitly discusses constraints related to scalability, real-world deployment, computational complexity, and generalization to unseen datasets. This section also outlines strategies for enhancing robustness and cross-domain applicability in future research

8. Is the proposed method computationally efficient enough for real-time or clinical application?

Response: Thank you for your comment. The proposed method demonstrates computational efficiency suitable for near–real-time clinical application, with inference times of approximately 1.563 s (non-tumor) and 3.175 s (tumor), confirming its feasibility for clinical use. We have added the answer in the Results and Discussion section, following the Processing Time Analysis (Table 2 and Fig. 8) subsection.

9. Rectify the minor errors observed in the paper through thorough copy editing. Improve the writing style, English language, and grammar.

Response: Thank you for your comment. We have corrected the grammatical in the manuscript.

10. Need to improve the conclusion section.

Response: Thank you for your comment. We have improved the conclusion section.
